# Safety and immunologic correlates of Melanoma GVAX, a GM-CSF secreting allogeneic melanoma cell vaccine administered in the adjuvant setting

**DOI:** 10.1186/s12967-015-0572-3

**Published:** 2015-07-05

**Authors:** Evan J Lipson, William H Sharfman, Shuming Chen, Tracee L McMiller, Theresa S Pritchard, January T Salas, Susan Sartorius-Mergenthaler, Irwin Freed, Sowmya Ravi, Hao Wang, Brandon Luber, Janice Davis Sproul, Janis M Taube, Drew M Pardoll, Suzanne L Topalian

**Affiliations:** Department of Oncology, Johns Hopkins University School of Medicine and Sidney Kimmel Comprehensive Cancer Center, 1550 Orleans Street, Room 507, Baltimore, MD 21287 USA; Department of Oncology, Johns Hopkins University School of Medicine and Sidney Kimmel Comprehensive Cancer Center, Baltimore, MD 21287 USA; Department of Surgery, Johns Hopkins University School of Medicine and Sidney Kimmel Comprehensive Cancer Center, Baltimore, MD USA; Department of Dermatology, Johns Hopkins University School of Medicine and Sidney Kimmel Comprehensive Cancer Center, Baltimore, MD USA; Division of Biostatistics and Bioinformatics, Department of Oncology, Johns Hopkins University School of Medicine and Sidney Kimmel Comprehensive Cancer Center, Baltimore, MD USA; Department of Pathology, Johns Hopkins University School of Medicine and Sidney Kimmel Comprehensive Cancer Center, Baltimore, MD USA

**Keywords:** Melanoma, Vaccine, Immunotherapy, Adjuvant, GM-CSF

## Abstract

**Background:**

Limited adjuvant treatment options exist for patients with high-risk surgically resected melanoma. This first-in-human study investigated the safety, tolerability and immunologic correlates of Melanoma GVAX, a lethally irradiated granulocyte–macrophage colony stimulating factor (GM-CSF)-secreting allogeneic whole-cell melanoma vaccine, administered in the adjuvant setting.

**Methods:**

Patients with stage IIB-IV melanoma were enrolled following complete surgical resection. Melanoma GVAX was administered intradermally once every 28 days for four cycles, at 5E7 cells/cycle (n = 3), 2E8 cells/cycle (n = 9), or 2E8 cells/cycle preceded by cyclophosphamide 200 mg/m^2^ to deplete T regulatory cells (Tregs; n = 8). Blood was collected before each vaccination and at 4 and 6 months after treatment initiation for immunologic studies. Vaccine injection site biopsies and additional blood samples were obtained 2 days after the 1st and 4th vaccines.

**Results:**

Among 20 treated patients, 18 completed 4 vaccinations. Minimal treatment-related toxicity was observed. One patient developed vitiligo and patches of white hair during the treatment and follow-up period. Vaccine site biopsies demonstrated complex inflammatory infiltrates, including significant increases in eosinophils and PD-1+ lymphocytes from cycle 1 to cycle 4 (P < 0.05). Serum GM-CSF concentrations increased significantly in a dose-dependent manner 48 h after vaccination (P = 0.0086), accompanied by increased numbers of activated circulating monocytes (P < 0.0001) and decreased percentages of myeloid-derived suppressor cells among monocytes (CD14+ , CD11b+ , HLA-DR low or negative; P = 0.002). Cyclophosphamide did not affect numbers of circulating Tregs. No significant changes in anti-melanoma immunity were observed in peripheral T cells by interferon-gamma ELIPSOT, or immunoglobulins by serum Western blotting.

**Conclusion:**

Melanoma GVAX was safe and tolerable in the adjuvant setting. Pharmacodynamic testing revealed complex vaccine site immune infiltrates and an immune-reactive profile in circulating monocytic cell subsets. These findings support the optimization of Melanoma GVAX with additional monocyte and dendritic cell activators, and the potential development of combinatorial treatment regimens with synergistic agents.

Trial registration: NCT01435499

**Electronic supplementary material:**

The online version of this article (doi:10.1186/s12967-015-0572-3) contains supplementary material, which is available to authorized users.

## Background

Several new drugs have been approved in recent years by the US Food and Drug Administration (FDA) for patients with unresectable and metastatic melanoma, but there remains a paucity of FDA-approved adjuvant therapies following melanoma resection. Interferon alfa (IFN-a) is a standard-of-care treatment for patients with high-risk stage IIB-III disease undergoing complete surgical resection. Although large clinical trials have consistently shown that IFN-a improves relapse free survival, there is inconsistent evidence regarding its impact on overall survival. For many patients, the toxicities of this year-long therapy outweigh the potential benefits [1–3]. Because patients with advanced primary melanomas or regional metastases have substantial 10-year melanoma-specific mortalities, ranging from 40 to 80%, more effective adjuvant therapies are sorely needed [4].

Immunotherapies such as high-dose interleukin-2 (IL-2), adoptive T cell transfer (ACT), and monoclonal antibodies (mAbs) blocking immune-inhibitory pathways (e.g., anti-CTLA-4, anti-PD-1) can induce durable objective tumor regressions in patients with advanced unresectable melanoma, attesting to the potency of anti-melanoma immunity in tumor rejection. [5–8] However, therapeutic melanoma vaccines administered in the advanced disease setting have yielded in vitro evidence of melanoma-specific immunization but only anecdotal clinical evidence of tumor regression [9, 10]. In the context of adjuvant therapy, a similar story has emerged, with various melanoma vaccines eliciting laboratory evidence of vaccine-specific immunity but no evidence of improved clinical outcomes [3, 11, 12]. However, vaccines capable of inducing melanoma-specific immune activation still hold promise for eliminating or stabilizing microscopic post-resection melanoma deposits.

In this first-in-human study, we investigated the safety, tolerability and immunologic correlates of Melanoma GVAX, a lethally irradiated granulocyte–macrophage colony stimulating factor (GM-CSF)-secreting allogeneic whole-cell melanoma vaccine which was administered in the adjuvant setting to patients with high-risk, surgically resected melanoma. In contrast to synthetic or recombinant vaccines targeting a particular antigen to stimulate anti-tumor immunity, Melanoma GVAX is a polyvalent vaccine derived from a cultured melanoma cell line expressing a plurality of shared tumor antigens. It is therefore theoretically capable of raising diverse immune responses recruiting CD4+ and CD8+ T cells, that may better immunize against the patient’s tumor and prevent resistance that might otherwise occur through the emergence of antigen-loss variants [13, 14]. Melanoma GVAX has been genetically modified to secrete GM-CSF, an immune-modulatory cytokine that can activate antigen presenting cells (APCs, monocytes and dendritic cells) locally at the vaccine site [15–17]. Indeed, autologous and/or allogeneic GM-CSF-secreting tumor cell vaccines have demonstrated evidence of clinical and/or in vitro immunologic responses in patients with various types of cancer [18–22]. Autologous melanoma GVAX formulations have been previously tested in the clinic. While the use of autologous tumor cells may preserve unique antigens expressed by each subject’s cancer, individual vaccine development requires laborious processing and regulatory testing, with the potential for disease progression to occur during the time required to generate the vaccine [18–22].

The vaccine strategy employed in the current study aimed to reduce immunosuppressive factors that might influence outcomes. Because excessive concentrations of GM-CSF have been shown to dampen immunity by expanding myeloid-derived suppressor cells (MDSCs) [23–26], Melanoma GVAX was designed to secrete moderate cytokine levels. Additionally, T regulatory cells (Tregs) may dampen antitumor immunity [27]. Murine and human tumors have been shown to induce the rapid expansion of CD4+CD25+ Tregs, impairing the rejection of otherwise immunogenic tumors [28]. Therefore, modest doses of cyclophosphamide (CPM), shown to elicit potent antitumor immunity in conjunction with tumor vaccines in animal models [29, 30], were administered in conjunction with Melanoma GVAX in one study cohort. We report here the clinical and immunological outcomes of this vaccine approach.

## Methods

### Melanoma GVAX

The melanoma cell line “526-mel”, initiated from a pulmonary metastatic melanoma lesion, was shown to express the common melanoma antigens tyrosinase, gp100 and MART-1/Melan-A, and MAGE-A3 by Western blotting and/or mRNA expression, and it was recognized by HLA-matched allogeneic CD4+ and CD8+ T cells specific for some of these antigens in vitro [10, 31–33]. Cultured cells were electroporated with a gene fragment encoding human GM-CSF and neomycin resistance factor. Subclone 526-5-6, secreting GM-CSF 200–400 ng/1E6 cells/24 h, was isolated and adapted to suspension culture to produce clinical vaccine lots of “Melanoma GVAX”. Melanoma GVAX cells were lethally irradiated prior to cryopreservation. Cells were thawed immediately prior to vaccine administration.

### Study design

The primary objective of this phase I trial was to evaluate the safety and feasibility of Melanoma GVAX, given with or without low dose CPM, in the adjuvant setting. Secondary objectives included pharmacodynamic assessments of GM-CSF effects and analysis of anti-melanoma immunization, including serologic and cellular responses. Consenting patients were enrolled in this study, approved by the IRB of the Sidney Kimmel Comprehensive Cancer Center at Johns Hopkins, between October 2011 and July 2013. Eligible patients had a confirmed histologic diagnosis of melanoma AJCC stage IIB–IV; had undergone surgical resection at least 2 weeks and no more than 6 months before starting protocol therapy; had no evidence of residual or recurrent tumor on physical examination or radiographic studies; had Eastern Cooperative Oncology Group (ECOG) performance status of ≤1; and had adequate hematologic, renal and hepatic function. Exclusion criteria included ocular melanoma, systemic melanoma therapy within 4 weeks, surgery or localized radiotherapy within 2 weeks, active or chronic infections including viral hepatitis or HIV, a history of autoimmune disease or immunodeficiency, any condition requiring systemic corticosteroids or other immunosuppressants, and prior immunotherapy including IFN-a and other cancer vaccines. A total of 20 research participants were sequentially enrolled in three treatment cohorts: Cohort A, low dose vaccine (5E7 cells/dose); Cohort B, high-dose vaccine (2E8 cells/dose); and Cohort C, high-dose vaccine with CPM 200 mg/m^2^ given intravenously one day prior to each vaccine. Treatment and biospecimen collection schedules are summarized in Additional file 1: Figure S1. Melanoma GVAX was administered in four 28-day cycles, by intradermal injection into the upper thighs or non-dominant upper arm, avoiding limbs involved in prior lymph node biopsy/dissection. Multiple spatially distributed skin sites were inoculated to provide optimal immunization [34–36]. Patients were assessed for vaccine-related toxicities and immunologic parameters until evidence of melanoma recurrence, or for a maximum of 6 months following treatment initiation. All patients were encouraged to enroll in a companion 5-year long-term follow-up protocol as required by the FDA for gene transfer treatment modalities. Participants leaving the study before receiving the second vaccination for reasons other than a dose-limiting toxicity (DLT) were replaced. Patients with relapsed melanoma were withdrawn from the study.

### Clinical assessments

Patients underwent radiographic evaluation, including CT scans of the body and MRI of the brain, before commencing treatment and at the 6-month visit (Additional file 1: Figure S1). Patients with resected stage IV disease were also assessed radiographically at 3 months. Medical history and physical examination, complete blood counts, comprehensive chemistry profile, serum lactate dehydrogenase and urinalysis were evaluated at baseline and prior to each treatment cycle. Complete blood counts were repeated on day 7 of each treatment cycle for patients receiving CPM (Cohort C). Adverse events were graded according to the National Cancer Institute Common Toxicity Criteria (CTC) version 4.0. Because the expected 10-year mortality in this study population is 40–80%, occurrence of grade 3–4 toxicities in up to 16% of patients in each cohort was deemed acceptable.

### Vaccine site biopsies

Vaccine site punch biopsies, 4 mm in diameter, were obtained 2 days after administration of the first and fourth vaccines, based on findings from published studies of autologous GM-CSF secreting melanoma vaccines [21, 22]. Formalin-fixed paraffin-embedded (FFPE) specimens were stained with hematoxylin and eosin (H&E), and immunohistochemical (IHC) analyses for CD1a, CD3, CD4, CD8, CD20, CD68, Fox-P3 and HMB-45 were performed by standard automated methods. PD-1 staining was done as described [7]. Histologic patterns of immune cell infiltration in the dermis and subcutaneous tissue and immune cell subsets were scored on a semi-quantitative scale. CD3 (T cell) and CD68 (macrophage) immunostains were scored as follows: 0, none; 1, rare scattered cells; 2, early perivascular infiltrate; 3, well-developed perivascular infiltrate; 4, perivascular infiltrate plus early interstitial infiltrate; 5, well-developed perivascular and interstitial infiltrate. Eosinophils were scored on H&E staining as: 0, none; 1, rare scattered cells; 2, perivascular; 3, perivascular and interstitial; 4, microabscesses (i.e., foci of >20 cells in one high power field); 5, sheet-like infiltrate of eosinophils with flame figures. Neutrophils were scored on H&E as: 0, absent; 1, scattered; 2, microabscesses. The CD4:CD8 ratio was established by IHC as 1:4, 1:2, 1:1, 2:1, 4:1, etc. CD20 B lymphocytes and CD1a Langerhans cells were scored as follows: 0, absent; 1, singular cells; 2, microabscesses; 3, germinal center formation. PD-1+ and Fox-P3+ lymphocyte subsets were scored as: 0, absent; 1, <5% of lymphocytes demonstrating expression; 2, 5–50% of lymphocytes demonstrating expression; 3, >50% lymphocytes demonstrating expression. The presence of Melanoma GVAX cells, and their expression of the melanoma antigen gp100 (IHC with mAb HMB-45), were graded according to the scale used for CD20 and CD1a.

### Serum GM-CSF

Serum was isolated from peripheral blood with BD Vacutainer SST tubes (Becton–Dickinson, Franklin Lakes, NJ, USA) and was cryopreserved at −80°C. GM-CSF was detected with the Quantikine High Sensitivity ELISA kit (detection range 1–64 pg/ml, R&D Systems, Minneapolis, MN, USA) per manufacturer’s instructions.

### Monocytic cell populations

Blood was collected at treatment cycles 1 and 4, immediately prior to treatment (Day 1 for Cohorts A and B, or Day 0 for Cohort C) and 2 days following vaccination (Day 3), to analyze the potential systemic effects of Melanoma GVAX on circulating monocyte populations. Peripheral blood mononuclear cells (PBMCs) were isolated by density gradient centrifugation (Lymphocyte Separation Medium, Lonza, Walkersville, MD, USA) and cryopreserved. All specimens from each patient were thawed and analyzed simultaneously. To assess effects on the numbers and activation state of circulating monocytes, PBMCs were stained with anti-HLA-DR, -CD14, -CD11b, or isotype-matched control mAbs (BD Biosciences, San Jose, CA, USA), and were analyzed by flow cytometry. The mean fluorescence intensity (MFI) of HLA-DR expression on CD14+CD11b+ events was analyzed as an indicator of monocyte activation. MDSCs were defined as FSC^hi^SSC^hi^CD14+CD11b+HLA-DR^lo^/(−), where the region of HLA-DR^lo^ monocytes was defined relative to gating on HLA-DR(−) lymphocytes. MDSCs were quantified as a percentage of CD14+CD11b+ monocytes, or by absolute number per μl of whole blood. Data were acquired on the BD FACSCalibur and analyzed using Flow Jo software (TreeStar, Ashland, OR, USA).

### T regulatory cells

To analyze the potential effects of CPM on circulating Tregs, PBMCs were co-stained with anti-CD4-FITC (RPA-T4, BD Biosciences, San Jose, CA, USA) and anti-CD25-PE (BC96, eBioscience, San Diego, CA, USA). Then, the FoxP3/Transcription factor staining buffer set (eBioscience) was used to assess intracellular expression of FoxP3 (anti-FoxP3-APC) according to manufacturer’s instructions. Tregs were characterized as CD4+CD25^hi^FoxP3+ lymphocytes, and were quantified as a percentage of circulating CD4+ T lymphocytes. Treg numbers in peripheral blood were also calculated by multiplying Treg frequencies in the lymphocyte population by absolute lymphocyte counts.

### Melanoma-specific T cell responses

Fresh pre- and post-vaccination PBMCs were thawed and incubated overnight in medium (RPMI 1640 + 10% heat-inactivated human AB serum), then tested for IFN-gamma (IFN-g) secretion in ELISPOT assays after stimulation with MHC class I (10 µM) or class II (20 µM) restricted melanoma-associated peptides, as described [37]. A pool of 32 CMV, EBV and influenza virus peptides was used as a positive control for T cell functionality (CEF, Cellular Technology Ltd, Shaker Heights, OH, USA). Alternatively, T cells were cultured in the presence of melanoma peptides for 10–13 days before ELISPOT, as follows [37]. PBMCs were depleted of CD4 or CD8 T cells using Dynabeads (Life Technologies, Carlsbad, CA, USA) according to the manufacturer’s protocol and were cultured at 1E6 cells/ml. CD4-depleted (CD8-enriched) cultures were stimulated with 10 µM MHC class I peptides, plus IL-7 and IL-15 (25 ng/ml each). In parallel, CD8-depleted (CD4-enriched) cultures were stimulated with 20 µM MHC class II peptides, plus GM-CSF 200 U/ml and IL-4 100 U/ml. Cultures were supplemented with IL-2 (10–120 IU/ml final concentration) starting at 24 h for CD4-depleted or 72 h for CD8-depleted cultures. Melanoma-associated peptides derived from tyrosinase, gp100, MART-1/Melan-A, phospho-MART-1, or MAGE-A3, and restricted by HLA-A1, -A2, -A3, -A24, -DR1, -DR13, or -DR15, were tested as appropriate for each patient’s HLA genotype (peptide sequences provided in Additional file 2: Table S1) [38–40]. Hepatitis B virus core antigen and influenza hemagglutinin peptides were used as negative MHC I and II controls, respectively, for short-term in vitro-stimulated ELISPOTs. Synthetic peptides (Pi Proteomics, Huntsville, AL, USA and Synthetic Biomolecules, San Diego, CA, USA) were certified to have >90% purity by analytical HPLC and mass spectroscopy (Tufts University Core Facility, Boston, MA, USA).

### Serum Western blots

To assess the potential for Melanoma GVAX to induce melanoma-specific IgG responses, serially collected sera were diluted 1:50 and used to probe Western blots containing lysates of 526-mel (parent line for Melanoma GVAX) or COS-7 cells (negative control), as described [38]. Blots were counterstained with peroxidase-conjugated goat anti-human IgG (Sigma, St. Louis, MO, USA). As a positive control for detecting melanoma antigens, blots were probed with the murine anti-human MART-1 mAb M2-7C10 (0.5 mg/ml; Covance, Emeryville, CA, USA).

### Statistical considerations

This study was designed to address its primary objectives to determine the toxicity and tolerability of Melanoma GVAX administered in the adjuvant setting, with or without low dose CPM. Thus, the trial was designed to include at least 19 patients in 3 sequentially enrolled cohorts (3 in Cohort A, 8 in Cohort B, and 8 in Cohort C). Changes in pharmacodynamic variables were evaluated using the Wilcoxon paired-sample signed rank test, and comparisons between cohorts were performed using the Mann–Whitney *U* test. Analyses of peripheral blood leukocyte subsets over time on treatment and among cohorts were performed using linear mixed effect models. Potential correlations between IFN-g serum concentrations and monocyte characteristics were subjected to a 2-sided Spearman correlation analysis. All statistical analyses were 2-sided, and p values <0.05 were considered significant (SAS software v.9.3, Cary, NC; R version 2.15.1; or GraphPad Prism v.5, San Diego, CA, USA). Secondary endpoints were considered exploratory and included changes in anti-melanoma immune responses. A positive immune response was defined as a two-fold increase in melanoma-specific reactivity compared to background assay values, comparing pre- to post-treatment levels.

## Results

### Patients

A total of 20 patients [8 female, 12 male; median age 55 years (range 22–75 years)] initiated treatment with Melanoma GVAX, including 3, 9, and 8 patients in Cohorts A, B, and C, respectively. Eighteen patients received all four vaccination cycles. One patient in Cohort B expired from unrelated causes after receiving two cycles of therapy. Another patient in Cohort B withdrew consent after receiving only one treatment cycle and was replaced. Patient characteristics are provided in Additional file 2: Table S2.

### Safety outcomes

Melanoma GVAX was well-tolerated, and there were no grade 3 or 4 treatment-related toxicities. Treatment-related adverse events are summarized in Additional file 2: Table S3.

Localized vaccine injection site reactions included erythema, induration, tenderness, swelling and pruritus. Except for one instance of grade 2 erythema, and one instance of grade 2 pruritus, these reactions were grade 1 in severity and resolved spontaneously or with the use of topical aloe cream.

Treatment-related systemic adverse events were predominantly grade 1 and included fatigue and flu-like symptoms. These symptoms resolved without intervention or with the use of over-the-counter non-steroidal anti-inflammatory agents. Isolated instances of grade 2 adverse events included dyspepsia, fatigue, and rash (1 patient each). One patient in Cohort A developed vitiligo and patches of white hair (described below). One patient in Cohort B who withdrew consent after Cycle 1 reported increased swelling of a non-vaccinated extremity which was the site of a prior lymphadenectomy, although clinical assessment revealed no change in mild baseline lymphedema. In Cohort C, 5 of 8 patients receiving low-dose CPM reported grade 1–2 nausea and/or dyspepsia, compared with none of 12 patients in cohorts A and B without CPM. No patient in Cohort C developed neutropenia or lymphopenia 7 days after receiving low-dose CPM in any cycle: total white blood cell counts ranged from 3,800 to 8600/cu mm (normal 3,500–11,000), and the lowest absolute lymphocyte count observed was 1,600/cu mm (normal 1,100–3,500).

At the 1-year observation interval on a companion long-term follow-up protocol, all drug-related adverse reactions had resolved and no new toxicities had developed.

### Tumor assessments

**Figure 1:**
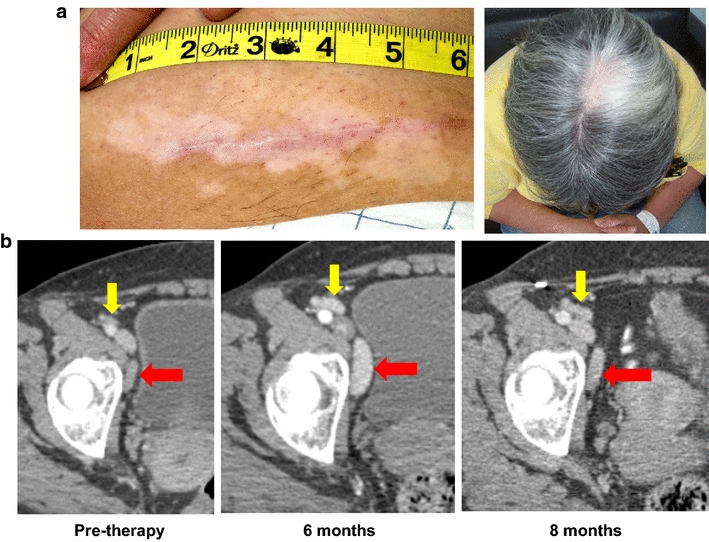
Transient melanoma progression in a patient treated in Cohort A, accompanied by vitiligo. **a** This patient developed the onset of vitiligo and greying hair after receiving four cycles of Melanoma GVAX. Vitiligo was initially confined to a prior lymphadenectomy incision site, but progressed to other skin sites and hair over the next few months. **b** Pelvic CT scan at the 6-month evaluation interval showed enlarging left pelvic lymph nodes (*yellow* and *red arrows*), biopsy-proven to be recurrent melanoma. At 8 months, these lesions had regressed without further intervention. Two months later, they enlarged again and the patient went on to receive other therapies.

Among 19 evaluable patients, 3 had a documented melanoma recurrence during the 6-month study period. One patient in Cohort A with resected stage III acral melanoma developed an inguinal lymph node recurrence at 6 months. On repeat CT scanning at 8 months, these lesions had regressed without further intervention, but at 10 months progression was confirmed. Of interest, this patient developed vitiligo and patches of white hair during the treatment and follow-up periods (Figure 1). A patient in Cohort B with resected stage IV melanoma developed a pulmonary metastatic recurrence. A patient in Cohort C with resected stage III melanoma developed tumor recurrence in the skin surrounding a prior melanoma excision site. The remaining 16 patients showed no signs of melanoma recurrence during the 6-month study period. All patients have been monitored for at least 14 additional months under a long-term follow-up companion protocol. Four patients—one in Cohort A, two in cohort B and one in Cohort C—have experienced melanoma recurrence between 7 and 33 months after treatment initiation. Tumor recurrence in a total of 7 of 19 patients to date is consistent with general expectations for this high-risk group.

### Vaccine site reactions

**Figure 2:**
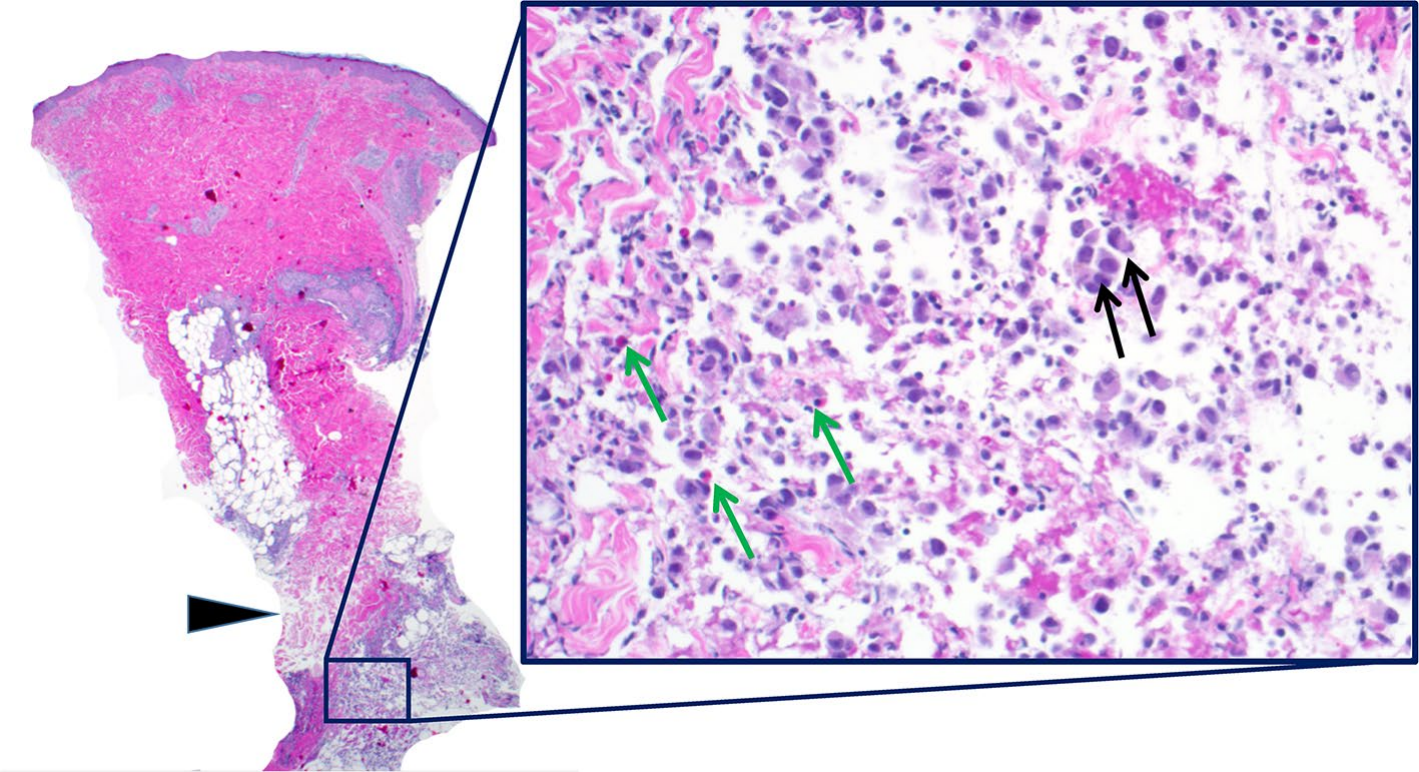
Vaccine site biopsy reveals a mixed inflammatory infiltrate. Representative photomicrograph of a skin punch biopsy obtained from a patient in Cohort A, 2 days after receiving the first dose of Melanoma GVAX. H&E staining reveals a dense, mixed inflammatory infiltrate centered primarily in the superficial subcutaneous adipose tissue (*blue inset box*), including lymphocytes, histiocytes, eosinophils, and irradiated melanoma vaccine cells. *Left image*, ×10 original magnification; right image, ×200 original magnification. *Black arrowhead*, dermal/subcutaneous junction; *black arrows*, Melanoma GVAX cells; *green arrows*, eosinophils. Immunohistochemical characterization is shown in Additional file 3: Figure S2.

Vaccine site biopsy specimens taken 2 days after both Cycle 1 and Cycle 4 vaccinations were available from 16 patients for CD3, CD4, CD8, CD20, and CD68 IHC. Because of limited material, H&E staining was performed on only 13 paired specimens, CD1a staining on 15, PD-1 and HMB-45 on 12, and FoxP3 on 8 paired specimens. A representative vaccine site biopsy is shown in Figure 2, and representative IHC stains are shown in Additional file 3: Figure S2, revealing selective infiltration of macrophages and eosinophils at vaccine sites. This is consistent with the local biological effects of GM-CSF secretion by Melanoma GVAX.

#### Treatment cycle effect

**Figure 3:**
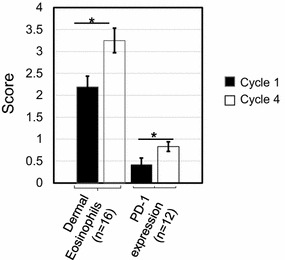
Vaccine site immune cell changes during treatment. Significant increases in the mean scores of dermal eosinophils and lymphocyte PD-1 expression from C1 to C4 were observed in Melanoma GVAX vaccine site biopsies. Paired analyses performed using the Wilcoxon signed-rank test; *asterisks* indicate p < 0.05. Scoring criteria are described in “Methods”. *Vertical bars* depict SEM. *C* treatment cycle.

When inflammatory infiltrates were compared in vaccine site skin biopsies from C1 vs. C4, a significant increase in the intensity of eosinophils was observed in C4, specifically in the dermal compartment (p = 0.024; Figure 3). Within the subcutaneous compartment, an increase in eosinophils from a mean grade of 3 to a mean grade of 4 was observed from cycle 1 to cycle 4, but this difference was not statistically significant. Furthermore, differences in neutrophil infiltrates were not observed when analyzed by treatment cycle or by compartment (data not shown). IHC staining of the same specimens revealed a significant increase in PD-1+ lymphocytes from C1 to C4 (p = 0.037, Figure 3), consistent with a vaccine-specific recall response. No significant changes were observed in the densities of CD3+ , CD4+ , CD8+ , or FoxP3+ T cells, CD20+ B cells, CD68+ macrophages, or CD1a+ Langerhans cells (data not shown).

#### Dose effect

Inflammatory vaccine site infiltrates were compared according to vaccine dose, low (Cohort A, 3 patients) vs. high (Cohorts B and C, 16 patients). Despite the small size of Cohort A, a significant increase in Langerhans cells was associated with the higher vaccine dose in C1 (low dose, mean score = 0, vs. high dose, mean = 0.73; p = 0.025, data not shown). An increase in PD-1+ lymphocytic infiltrates was associated with the higher vaccine dose at C4 (low dose, mean score = 0, vs. high dose, mean = 1; p = 0.012, data not shown). Trends (i.e., p < 0.1) suggested possible increases in dermal eosinophils and neutrophils with the higher vaccine dose in C1 and C4, respectively. A borderline decrease in the lymphohistocytic infiltrate in the subcutis was associated with higher vaccine dose at C1, along with a trend for an increasing CD4:CD8 ratio. There were no other significant dose-dependent histologic findings.

#### Cyclophosphamide effect

To determine the potential impact of CPM on the vaccine site immune milieu, inflammatory cell populations in the dermis and subcutis were compared for patients receiving the high dose vaccine, without CPM (Cohort B, 9 biopsy pairs) or with CPM (Cohort C, 7 biopsy pairs). Vaccine site biopsies demonstrated a significant increase in Langerhans cell density at C1 in patients who received CPM (p = 0.042; data not shown). No significant differences were seen among eosinophils, neutrophils, CD3+ , CD20+ , CD68+ , or PD-1+ cells, or CD4:CD8+ T cell ratios, in C1 or C4. Importantly, no differences in the intensity of FoxP3+ Tregs in vaccine site infiltrates were observed between patients receiving or not receiving CPM prior to each vaccine dose.

### Pharmacokinetics of GM-CSF

**Figure 4:**
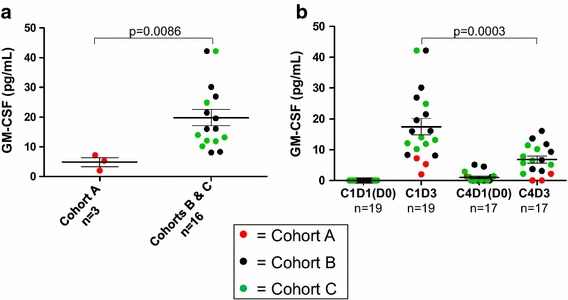
Melanoma GVAX administered intradermally increases systemic GM-CSF levels in a dose-dependent manner. **a** Serum GM-CSF concentrations measured 2 days after the first administration of Melanoma GVAX were significantly higher in patients receiving a dose of 2E8 cells (Cohorts B and C, mean 19.8 ± 2.72) compared to 5E7 cells (Cohort A, mean 4.8 ± 1.5). There was no significant difference between patients receiving high-dose vaccine in Cohorts B (no CPM) versus C (with CPM) (not shown). All patients had detectable serum GM-CSF after the first vaccination (detection limit 1 pg/ml). **b** Serum GM-CSF concentrations were significantly lower 2 days after the fourth vaccine (6.8 ± 1.5) compared to the first vaccine (17.5 ± 2.6). Bars depict the mean ± SEM; p values from 2-sided Mann–Whitney *U* test (**a**) or paired Wilcoxon signed-rank test (**b**). *C* treatment cycle, *D* treatment day. Pre-vaccine sera from Cohort C were collected on D0 prior to CPM administration, and from Cohorts A and B, on D1 prior to vaccine administration.

GM-CSF secretion from Melanoma GVAX cells injected intradermally was detectable systemically in all patients. Based on serum GM-CSF kinetics reported in trials of GVAX vaccines in other cancer types [41–43], we analyzed patient sera collected 2 days following the first and fourth vaccinations. Pre-treatment GM-CSF concentrations were below the limit of detection for all patients, but increased significantly and in a dose-dependent manner 2 days after the first vaccination (p = 0.0086). This increase was less pronounced after C4 compared to C1 (p = 0.0003) (Figure 4), suggesting more rapid elimination of allogeneic Melanoma GVAX with repeated inoculations [43].

### Peripheral blood leukocyte subsets

Complete blood counts and automated differentials were obtained prior to each vaccine cycle (i.e., at 1-month intervals), and at the 4- and 6-month assessments after treatment initiation. These data were analyzed for changes in leukocyte subset cell numbers and proportions over time on treatment, as well as comparisons among the three treatment cohorts (not shown). We observed significant decreases in lymphocyte numbers and percentages (p = 0.007 and 0.018, respectively; Linear mixed effect model) in the total treatment population over the 6-month study period, although most values remained within the normal range (Additional file 4: Figure S3). In contrast, numbers and percentages of neutrophils, monocytes, eosinophils, and basophils demonstrated no significant trends over time on treatment.

### Characterization of circulating monocytic cell populations

**Figure 5:**
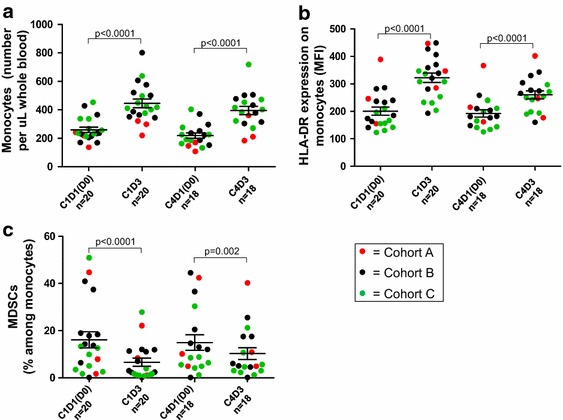
Melanoma GVAX coordinately increases numbers of activated circulating monocytes and decreases circulating MDSCs. Flow cytometric analysis revealed significantly increased numbers (**a**) and activation (**b**) of circulating monocytes (CD14+ , CD11b+) 2 days following the first and fourth vaccinations. Monocyte activation was quantified as mean fluorescence intensity (MFI) of HLA-DR expression. Decreased percentages of myeloid-derived suppressor cells (CD14+ , CD11b+ , HLA-DR low or negative) among monocytes were observed at the same time intervals (**c**). *Bars* depict the mean ± SEM; p values from 2-sided Wilcoxon signed-rank test. *MDSC* myeloid derived suppressor cells, *C* treatment cycle, *D* treatment day.

**Figure 6:**
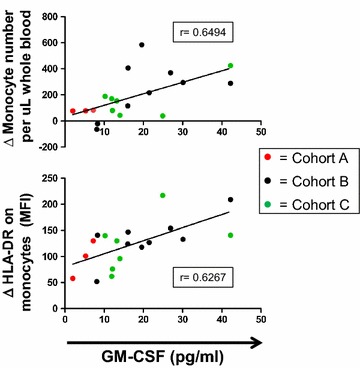
Changes in monocyte numbers and activation state 2 days following the first dose of Melanoma GVAX correlate with serum GM-CSF concentrations at C1D3. Changes (Δ) were calculated by subtracting baseline values (C1D1 for cohorts A and B, or C1D0 for Cohort C) from values obtained at cycle 1, day 3. Data from 19 patients are shown. Monocyte activation was quantified by measuring mean fluorescence intensity (MFI) of HLA-DR expression on CD14+ , CD11b+ events by flow cytometry. r values are from a 2-sided Spearman Correlation Analysis.

Because the acute biologic effects of GM-CSF on myeloid cell populations can be dose-dependent, with excessive doses generating MDSCs rather than activating antigen presenting cells [23], we evaluated the impact of Melanoma GVAX on the absolute numbers and proportions of circulating monocytes and MDSCs in blood collected before and 2 days after the first and fourth Melanoma GVAX treatment cycles. We observed significantly increased numbers and activation (HLA-DR expression) of circulating monocytes after vaccination (p < 0.0001) (Figure 5a, b, respectively). Additionally, decreased percentages of MDSCs (CD14+ , CD11b+ , HLA-DR low or negative) among monocytes were seen (p = 0.002) (Figure 5c). Serum GM-CSF levels measured 2 days following the first dose of Melanoma GVAX (cycle 1, day 3) correlated significantly with concurrent changes in monocyte numbers and activation state (HLA-DR expression) in 19 patients assessed (Figure 6). Thus, the amount of GM-CSF secreted by Melanoma GVAX (200–400 ng/1E6 cells/24 h in vitro) appeared to have a positive systemic effect on the balance between activated monocytes and MDSCs.

### Regulatory T cells (Tregs)

Cyclophosphamide (CPM) administered at low doses has been reported to selectively deplete circulating Tregs within 3 days after administration [44]. However, while it is possible that subtle differences in our study were obscured by small group size, we did not observe significant differences in numbers of circulating Tregs (CD4+ , CD25^hi^, FoxP3+) (not shown) or percentages of Tregs among CD4 + T cells, comparing blood samples collected from patients in Cohort C just prior to or 3 days after receiving CPM (Additional file 5: Figure S4). Furthermore, no significant fluctuations in Tregs were observed in patients who did not receive CPM (Cohorts A and B, Additional file 5: Figure S4).

### Assessments of systemic anti-melanoma immunity

**Figure 7:**
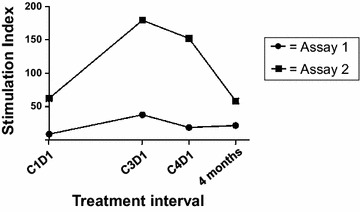
Increased melanoma peptide-specific reactivity generated in one patient over time on treatment with Melanoma GVAX. This patient in Cohort A was HLA-A2+ . IFN-g ELISPOT was used to measure the specific reactivity of short-term in vitro stimulated T cell cultures against a pool of melanoma peptides restricted by HLA-A2. Stimulation index = [IFN-g spot forming colonies/1E6 cells stimulated with melanoma peptide pool]/[IFN-g spot forming colonies/1E6 cells stimulated with HBV control peptide]. Trends were similar in two separate assays, showing peak reactivity after two cycles of treatment. *C* treatment cycle; *D* treatment day.

In patients with appropriate HLA genotypes, serially collected PBMCs were assessed for CD8+ and CD4+ T cell reactivity against commonly expressed melanoma epitopes restricted by HLA class I and II molecules, respectively (Additional file 2: Table S1). Source proteins for melanoma epitopes included tyrosinase, MART-1, gp100, and MAGE-A3, all known to be expressed by Melanoma GVAX. Individual synthetic peptides or peptide pools were tested as appropriate to each patient’s HLA genotype. Two patients did not have HLA class I, and four did not have HLA class II types which were amenable to testing. In 18 patients tested (3 from Cohort A, 9 from Cohort B, 6 from Cohort C), fresh uncultured T cells did not show specificity for melanoma peptides at baseline (C1D1, or C1D0 for Cohort C) or at any of the subsequent time points tested [C2D1(D0), C3D1(D0), C4D1(D0)), and 4 months post-treatment initiation], using a highly sensitive IFN-g ELISPOT assay. However, in the same assays, fresh lymphocytes from 17/18 patients demonstrated robust reactivity against viral recall antigens (CMV, EBV, influenza virus) (mean 738–893 IFN-g spot forming colonies per 1E6 PBMCs), providing a positive control that did not vary significantly in amplitude over the course of treatment (not shown). Following short-term cultures with melanoma peptide stimulation, T cells from 18 patients were re-assessed for melanoma peptide recognition in ELISPOT assays. Among 18 patients tested, eight were HLA-A2+ , and all eight demonstrated specific reactivity against a pool of A2-restricted melanoma peptides. Only 6 of 8 HLA-A2+ patients had both pre- and post- treatment blood samples available for testing; among them, all showed anti-melanoma reactivity at baseline. Reactivity increased following vaccination in 1 of 6 patients (Figure 7), while 5 of 6 patients had decreasing or variable anti-melanoma reactivity over time on treatment. No reactivity was detected in any patient against peptides restricted by non-A2 HLA molecules (data not shown).

Using serially collected serum samples, 18 patients receiving Melanoma GVAX were also assessed for IgG responses against the vaccine, using a serum Western blotting technique. [38] Analysis of serially collected specimens did not reveal the development or enhancement of specific IgG reactivity against proteins expressed by Melanoma GVAX over the course of treatment (not shown).

## Discussion

Cancer vaccines hold promise for augmenting specific antitumor immunity above a threshold needed for tumor elimination. However, many studies of vaccine monotherapy conducted in patients with advanced cancers have failed to produce significant response rates [10]. An important question is whether such vaccines might be more effective in the setting of microscopic or “minimal residual” disease. Accordingly, we undertook this first-in-human study of a novel melanoma vaccine in patients who had no clinical evidence of disease following surgery but were predicted to be at high risk for recurrence due to the clinicopathologic characteristics of their tumors. The administration of the Melanoma GVAX vaccine proved to be feasible and safe. The successful administration of this allogeneic whole cell vaccine to all participants in this trial distinguishes it from autologous GM-CSF-transduced melanoma vaccines which are more variable and difficult to produce. For example, in a study of an autologous GM-CSF-secreting melanoma cell vaccine by Luiten and colleagues in 64 subjects with advanced disease, only 28 patients received the full treatment regimen due to the complexities of tumor cell processing and the time required for vaccine production [22].

Recent research has emphasized the importance of individually mutated tumor antigens in antitumor immune responses mediating tumor rejection, especially in the context of monoclonal antibody therapies blocking immune “checkpoints” such as CTLA-4 and PD-1 [45]. However, there is also evidence supporting the rationale for an allogeneic vaccine approach generating anti-melanoma immunity against shared tumor antigens. Several of the tumor antigens that have been identified in melanoma are shared among >50% of melanoma patients [46, 47]. These can be effective tumor rejection antigens, as shown in clinical trials of adoptive T cell transfer targeting MART-1/MelanA, gp100 and NY-ESO-1 [48, 49]. Recent trials of allogeneic GM-CSF-secreting tumor cell vaccines in non-melanoma cancers have demonstrated an association of T cell and immunoglobulin responses raised against shared tumor antigens, with favorable clinical outcomes. A phase 2 randomized trial of pancreatic cancer GVAX administered with or without a live-attenuated *Listeriamonocytogenes* immunotherapy (CRS-207) demonstrated extended survival in patients receiving the vaccine combination compared to pancreatic GVAX alone; this was associated with the induction of mesothelin-specific CD8+ T lymphocytes [50]. Similarly, development of a prostate-specific membrane antigen (PSMA)-specific antibody response was associated with improvement in overall survival after treatment with prostate cancer GVAX combined with ipilimumab (anti-CTLA-4) [51].

The administration of GM-CSF in certain clinical settings has provided evidence for anti-melanoma activity. A randomized phase 2 trial comparing ipilimumab plus recombinant GM-CSF to ipilimumab alone in 245 patients with unresectable stage III or IV melanoma demonstrated an improved one-year overall survival rate (68.9 vs 52.9%; P_1_ = 0.01) and decreased toxicity in the combination treatment arm [52]. Talimogene laherparepvec (T-VEC, formerly Oncovex^GM-CSF^), an attenuated oncolytic herpesvirus engineered to encode human GM-CSF, was demonstrated to improve overall response rates when injected intratumorally, compared to subcutaneous recombinant GM-CSF administration in a randomized phase 3 study of 436 patients with advanced melanoma (26.4 vs 5.7%, P < 0.0001) [53]. However, pharmacodynamic evidence for the local or systemic biological effects of GM-CSF has not been reported in these trials. In the current trial of Melanoma GVAX, we monitored serum GM-CSF levels and demonstrated increased numbers of activated circulating monocytes concomitant with a reduction in MDSCs, a favorable balance for immune activation [26, 54]. Serum GM-CSF levels were significantly lower after the fourth compared to the first vaccination, suggesting that GVAX vaccine cells were cleared more rapidly after the fourth vaccine by a primed anti-alloantigen immune response. A similar phenomenon was reported in a trial of an allogeneic GM-CSF-secreting breast tumor vaccine [43].

The administration of GM-CSF–secreting tumor cell vaccines combined with anti-CTLA-4 has been associated with an increased ratio of intratumoral CD8+ T lymphocytes to Tregs [55, 56]. Because our study was conducted in the adjuvant setting, we were unable to assess vaccine-induced changes in the tumor microenvironment. However, we did evaluate immunologic phenomena at Melanoma GVAX vaccination sites after the first and fourth vaccinations, and found increased PD-1 expression after the fourth vaccine. This suggests local lymphocyte activation and the potential for enhancing the effectiveness of Melanoma GVAX in raising antitumor immunity by incorporating anti-PD-1 into the treatment regimen.

Low-dose cyclophosphamide was assessed in Cohort C of this trial as a potential immune enhancer, because of evidence showing that it may augment vaccine-induced antigen-specific T cell responses and decrease the numbers and functionality of Tregs [57, 58]. Emens and colleagues reported a dose-ranging study of CPM, doxorubicin, and an allogeneic GM-CSF-secreting breast cancer vaccine in 28 patients with metastatic disease [43]. HER2-specific antibody responses were enhanced by 200 mg/m^2^ CPM, whereas higher doses of CPM suppressed immunity. In contrast to that study and others suggesting immunopotentiation from low-dose CPM [59, 60], results from our study demonstrated no effect of CPM on circulating or vaccine site Tregs, or on systemic anti-melanoma T or B cell immunity. Because patients on this trial had no clinical evidence of melanoma at the time of enrollment, we were unable to assess effects that CPM might have had on immune infiltrates in residual microscopic tumor deposits.

## Conclusion

In conclusion, Melanoma GVAX is safe when administered in the adjuvant setting to patients with high-risk resected melanoma. Biological activity is suggested by complex vaccine site immune infiltrates and an immune-reactive profile in circulating monocyte subsets. PD-1 expressing lymphocytes were observed at vaccine inoculation sites. Taken together, these findings support the optimization of Melanoma GVAX with additional monocyte and dendritic cell activators, and the potential development of combination treatment regimens with PD-1 blocking drugs.

## Additional files

Additional file 1:
**Figure S1.** Schedule of Melanoma GVAX treatment, biospecimen collection and clinical assessment. Asterisks denote vaccine administration (Day 1 of each cycle). Cyclophosphamide was administered one day prior to each vaccine administration for patients in cohort C. Peripheral blood (PB) was collected at baseline (C1D1, or C1D0 for Cohort C) and prior to each vaccination [C2D1(D0), C3D1(D0) and C4D1(D0)], Treatment cycle length is 28 days. C, treatment cycle; D, treatment day. Additional file 2:
**Table S1.** Peptide sequences used to assess T cell responses. **Table S2.** Patient characteristics. **Table S3.** Treatment-related adverse events. Additional file 3:
**Figure S2.** Immune cells infiltrating a Melanoma GVAX vaccine site biopsy are predominantly macrophages. Representative skin biopsy obtained from a patient in Cohort A, 2 days after receiving Cycle 1 of Melanoma GVAX. IHC staining demonstrates a mixed inflammatory infiltrate composed primarily of CD68+ macrophages/histiocytes, with accompanying CD3+ T cells. Melanoma GVAX vaccine cells express the gp100 antigen in situ, as shown by HMB-45 staining. Only rare CD20+ B cells were observed. 200× original magnification, all fields. Additional file 4:
**Figure S3.** Trends in peripheral lymphocyte numbers and percentages over time on treatment. Significant decreases in mean peripheral lymphocyte numbers (left panel, p = 0.007) and percentages (right panel, p = 0.018) occurred over time on treatment and follow-up. Linear mixed effect model was used for comparisons. Mean values, represented by solid circles, are connected by a trendline in each graph. Rectangles at each time point extend to the 1st and 3rd quartiles. Vertical lines at each time point extend to 1.5 x the interquartile range. Dashed lines indicate upper and lower limits of normal values. Each treatment cycle length is 28 days. Samples were obtained on Day 1 of each cycle for patients in Cohorts A and B, and on Day 0 of each cycle for patients in Cohort C. An analysis including all patients is shown. Similar trends were seen for each cohort when analyzed individually (data not shown). C, treatment cycle. Additional file 5:
**Figure S4.** Peripheral blood Treg percentages are not significantly affected by the administration of low-dose CPM prior to Melanoma GVAX inoculation. Right panel: no significant change in the percentage of Tregs (CD4+ , CD25^hi^, FoxP3 +) among circulating CD4+ T lymphocytes was observed 3 days following CPM 200 mg/m^2^ IV, in vaccination cycles 1 or 4. Left panel: Treg percentages before and after Melanoma GVAX administration without CPM, in Cohorts A and B. Patients in Cohort C received CPM at D0, and Melanoma GVAX at D1 of each cycle. Bars depict the mean ± SEM. Comparisons were not significant using a paired Wilcoxon signed-rank test. C, treatment cycle; D, treatment day.

## Abbreviations

ACT: adoptive T cell transfer
AJCC: American Joint Committee on Cancer
APC: antigen presenting cell
CPM: cyclophosphamide
CTC: common toxicity criteria
CTLA-4: cytotoxic T lymphocyte-associated protein 4
DLT: dose-limiting toxicity
ECOG: Eastern Cooperative Oncology Group
FDA: US Food and Drug Administration
GM-CSF: granulocyte–macrophage colony-stimulating factor
H&E: hematoxylin and eosin
HLA: human leukocyte antigen
IFN-a: interferon alfa
IFN-g: interferon gamma
IHC: immunohistochemistry
IL-2: interleukin-2
mAbs: monoclonal antibodies
MDSC: myeloid-derived suppressor cells
MFI: mean fluorescence intensity
PBMCs: peripheral blood mononuclear cells
PD-1: programmed death-1
PSMA: prostate-specific membrane antigen
Tregs: T regulatory cells
