# Supplementary material for: Safety and immunologic correlates of Melanoma GVAX, a GM-CSF secreting allogeneic melanoma cell vaccine administered in the adjuvant setting
Source: J Transl Med. 2015 Jul 5;13:214. doi: 10.1186/s12967-015-0572-3 (PMC4491237; doi:10.1186/s12967-015-0572-3)
Supplement: Additional file 1: — Figure S1. Schedule of Melanoma GVAX treatment, biospecimen collection and clinical assessment. Asterisks denote vaccine administration (Day 1 of each cycle). Cyclophosphamide was administered one day prior to each vaccine administration for patients in cohort C. Peripheral blood (PB) was collected at baseline (C1D1, or C1D0 for Cohort C) and prior to each vaccination [C2D1(D0), C3D1(D0) and C4D1(D0)], Treatment cycle length is 28 days. C, treatment cycle; D, treatment day. [file 12967_2015_572_MOESM1_ESM.pptx]

## Slide 1
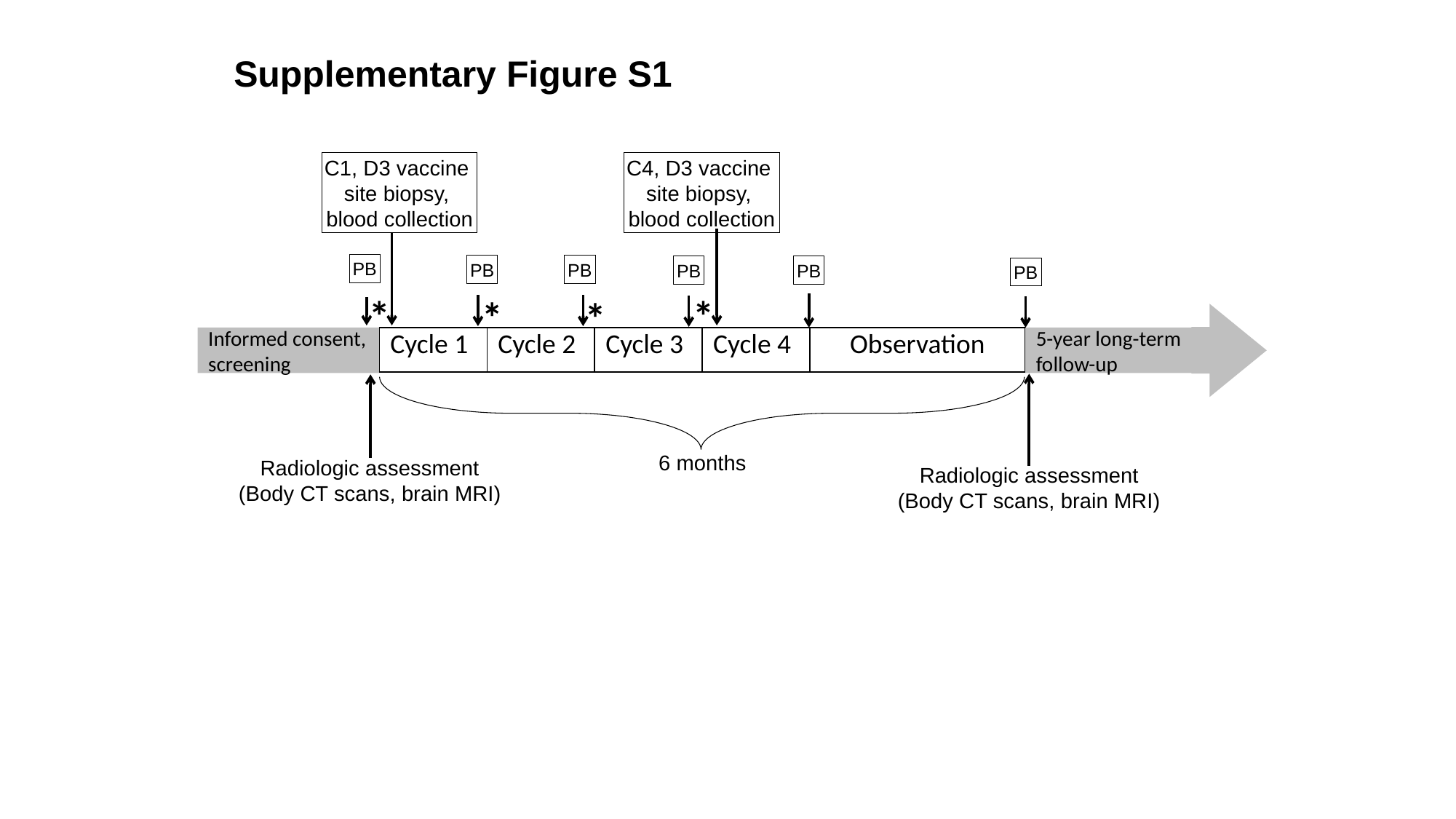

Supplementary Figure S1
C1, D3 vaccine
site biopsy,
blood collection
C4, D3 vaccine
site biopsy,
blood collection
PB
PB
PB
PB
PB
PB
*
*
*
*
| Cycle 1 | Cycle 2 | Cycle 3 | Cycle 4 | Observation |
| --- | --- | --- | --- | --- |
5-year long-term follow-up
Informed consent, screening
6 months
Radiologic assessment
(Body CT scans, brain MRI)
Radiologic assessment
(Body CT scans, brain MRI)
