# Supplementary material for: Safety and immunologic correlates of Melanoma GVAX, a GM-CSF secreting allogeneic melanoma cell vaccine administered in the adjuvant setting
Source: J Transl Med. 2015 Jul 5;13:214. doi: 10.1186/s12967-015-0572-3 (PMC4491237; doi:10.1186/s12967-015-0572-3)
Supplement: Additional file 2: — Table S1. Peptide sequences used to assess T cell responses. Table S2. Patient characteristics. Table S3. Treatment-related adverse events. [file 12967_2015_572_MOESM2_ESM.docx]

**Supplementary Table S1: Peptide sequences**

| **Antigen** | **HLA Restriction** | **Residues** | | **Amino Acid Sequence** |
| --- | --- | --- | --- | --- |
| gp100 | A2 | 209-217, 210M | IMDQVPFSV | |
|  | A2 | 280-288, 288V | YLEPGPVTV | |
|  | A3 | 17-25 | ALLAVGATK | |
|  | DR4 | 44-59 | WNRQLYPEWTEAQRLD | |
| MAGE-A3 | DR13 | 121-134 | LLKYRAREPVTKAE | |
| MART-1/Melan-A | A2 | 26-35, 27L | ELAGIGILTV | |
|  | DR1 | 100-115 | APPAYEKLSAEQSPPP | |
| pMART-1/Melan-A | DR1 | 100-115 | APPAYEKLpSAEQSPPP | |
| Tyrosinase | A1 | 243-251, 244S | KSDICTDEY | |
|  | A2 | 369-377, 371D | YMDGTMSQV | |
|  | A24 | 206-214 | AFLPWHRLF | |
|  | DR4 | 56-70, 63V | QNILLSNVPLGPQFP | |
|  | DR15 | 386-406 | FLLHHAFVDSIFEQWLRRHRP | |
|  | DR15 | 386-406, 402Q | FLLHHAFVDSIFEQWLQRHRP | |
| *Hepatitis B virus core | A2 | 18-27, 27V | FLPSDFFPSV | |
| *Influenza hemagglutinin | DR1, DR4 | 307-319 | PKYVKQNTLKLAT | |
|  |  |  |  | |
| * Negative controls |  |  |  | |

**Supplementary Table S2: Patient characteristics**

|  | **Cohort A^a^; n=3** | **Cohort B^b^; n=9** | **Cohort C^c^; n=8** | **Total; N=20** |
| --- | --- | --- | --- | --- |
| **Age (yrs; median, range)** | 49.9 (22-53) | 62.0 (40-69) | 60.7 (42-75) | 55.2 (22-75) |
| **Gender (n, %)** |  |  |  |  |
| Male | 1 (33%) | 4 (44%) | 7 (88%) | 12 (60%) |
| Female | 2 (67%) | 5 (56%) | 1 (12%) | 8 (40%) |
| **Melanoma clinicopathologic stage^d^ (n, %)** |  |  |  |  |
| IIB | 1 (33%) | 2 (22%) | 1 (12%) | 4 (20%) |
| IIIA | 1 (33%) | 3 (33%) | 5 (63%) | 9 (45%) |
| IIIB | 0 | 1 (11%) | 1 (12%) | 2 (10%) |
| IIIC | 1 (33%) | 2 (22%) | 1 (12%) | 4 (20%) |
| IV | 0 | 1 (11%) | 0 | 1 (5%) |

^A^Vaccine alone (5E7 cells per dose)

^b^Vaccine alone (2E8 cells per dose)

^c^Vaccine (2E8 cells per dose) plus CPM (200mg/m2) given one day prior to vaccination

^d^All patients underwent complete surgical resection of tumor prior to enrolling in study

**Supplementary Table S3: Treatment-related adverse events**

| **Injection site reactions, by vaccine cycle** | |  |  |  |  |  |  |  |
| --- | --- | --- | --- | --- | --- | --- | --- | --- |
|  | **Cohort A (n=3)** | |  | **Cohort B (n=9)** | |  | **Cohort C (n=8)** | |
| **Cycle 1** | **Grade 1** | **Grade 2** |  | **Grade 1** | **Grade 2** |  | **Grade 1** | **Grade 2** |
| Erythema | 2 | 1 |  | 9 | 0 |  | 8 | 0 |
| Pruritus | 2 | 0 |  | 8 | 1 |  | 5 | 0 |
| Pain/soreness | 2 | 0 |  | 5 | 0 |  | 5 | 0 |
| Induration | 3 | 0 |  | 9 | 0 |  | 8 | 0 |
| Burning sensation | 0 | 0 |  | 9 | 0 |  | 8 | 0 |
|  | **Cohort A (n=3)** | |  | **Cohort B (n=8)** | |  | **Cohort C (n=8)** | |
| **Cycle 2** | **Grade 1** | **Grade 2** |  | **Grade 1** | **Grade 2** |  | **Grade 1** | **Grade 2** |
| Erythema | 3 | 0 |  | 8 | 0 |  | 8 | 0 |
| Pruritus | 2 | 0 |  | 6 | 0 |  | 6 | 0 |
| Pain/soreness | 1 | 0 |  | 3 | 0 |  | 4 | 0 |
| Induration | 3 | 0 |  | 7 | 0 |  | 8 | 0 |
| Burning sensation | 0 | 0 |  | 8 | 0 |  | 8 | 0 |
|  | **Cohort A (n=3)** | |  | **Cohort B (n=7)** | |  | **Cohort C (n=8)** | |
| **Cycle 3** | **Grade 1** | **Grade 2** |  | **Grade 1** | **Grade 2** |  | **Grade 1** | **Grade 2** |
| Erythema | 3 | 0 |  | 7 | 0 |  | 8 | 0 |
| Pruritus | 3 | 0 |  | 7 | 0 |  | 6 | 0 |
| Pain/soreness | 1 | 0 |  | 3 | 0 |  | 3 | 0 |
| Induration | 3 | 0 |  | 7 | 0 |  | 8 | 0 |
| Burning sensation | 0 | 0 |  | 7 | 0 |  | 8 | 0 |
|  |  |  |  |  |  |  |  |  |
|  | **Cohort A (n=3)** | |  | **Cohort B (n=7)** | |  | **Cohort C (n=8)** | |
| **Cycle 4** | **Grade 1** | **Grade 2** |  | **Grade 1** | **Grade 2** |  | **Grade 1** | **Grade 2** |
| Erythema | 3 | 0 |  | 7 | 0 |  | 8 | 0 |
| Pruritus | 3 | 0 |  | 7 | 0 |  | 5 | 0 |
| Pain/soreness | 2 | 0 |  | 4 | 0 |  | 5 | 0 |
| Induration | 3 | 0 |  | 7 | 0 |  | 8 | 0 |
| Burning sensation | 0 | 0 |  | 7 | 0 |  | 8 | 0 |
|  |  |  |  |  |  |  |  |  |
| **All other treatment-related adverse events, by system organ class** | |  |  |  |  |  |  |  |
|  | **Cohort A (n=3)** | |  | **Cohort B (n=9)** | |  | **Cohort C (n=8)** | |
| **Blood and Lymphatic system disorders** | |  |  |  |  |  |  |  |
| Lymph node swelling | 1 | 0 |  | 0 | 0 |  | 0 | 0 |
| **Gastrointestinal** |  |  |  |  |  |  |  |  |
| Nausea | 0 | 0 |  | 0 | 0 |  | 4 | 0 |
| Vomiting | 0 | 0 |  | 0 | 0 |  | 1 | 0 |
| Dysphagia | 0 | 0 |  | 1 | 0 |  | 0 | 0 |
| Constipation | 0 | 0 |  | 0 | 0 |  | 1 | 0 |
| Dyspepsia | 0 | 0 |  | 0 | 0 |  | 0 | 1 |
| **General Disorders** |  |  |  |  |  |  |  |  |
| Fatigue | 1 | 0 |  | 3 | 0 |  | 7 | 1 |
| Fever | 0 | 0 |  | 0 | 0 |  | 1 | 0 |
| Chills | 0 | 0 |  | 3 | 0 |  | 0 | 0 |
| Facial edema | 0 | 0 |  | 1 | 0 |  | 0 | 0 |
| Flu-like symptoms | 0 | 0 |  | 0 | 0 |  | 1 | 0 |
| Limb edema | 0 | 0 |  | 2 | 0 |  | 0 | 0 |
| **Musculoskeletal/connective tissue** |  |  |  |  |  |  |  |  |
| Limb weakness | 1 | 0 |  | 3 | 0 |  | 0 | 0 |
| Arthralgia | 0 | 0 |  | 1 | 0 |  | 1 | 0 |
| Myalgia | 1 | 0 |  | 2 | 0 |  | 1 | 0 |
| **Nervous system disorders** |  |  |  |  |  |  |  |  |
| Headache | 0 | 0 |  | 1 | 0 |  | 0 | 0 |
| **Skin and subcutaneous tissue disorders** |  |  |  |  |  |  |  |  |
| Pruritus | 0 | 0 |  | 1 | 0 |  | 0 | 0 |
| Vitiligo | 1 | 0 |  | 0 | 0 |  | 0 | 0 |
| Rash | 0 | 0 |  | 1 | 1 |  | 0 | 0 |
| **Vascular** |  |  |  |  |  |  |  |  |
| Flushing | 0 | 0 |  | 1 | 0 |  | 0 | 0 |
