# Supplementary figures and images for: Safety and immunologic correlates of Melanoma GVAX, a GM-CSF secreting allogeneic melanoma cell vaccine administered in the adjuvant setting
Source: J Transl Med. 2015 Jul 5;13:214. doi: 10.1186/s12967-015-0572-3 (PMC4491237; doi:10.1186/s12967-015-0572-3)

## Slide 1
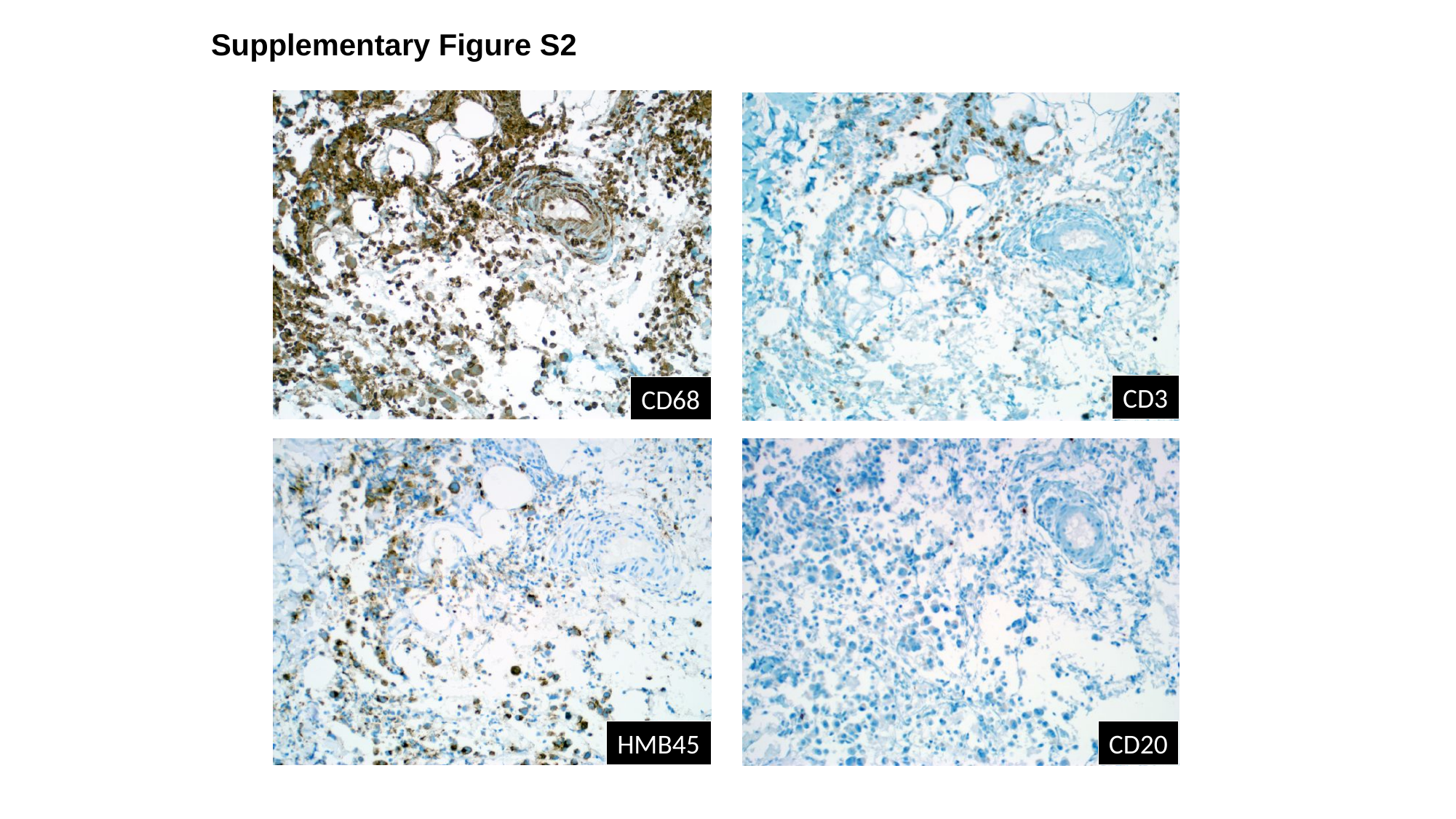

# Supplementary Figure S2
CD3
CD68
CD20
HMB45

Supplement: Additional file 3: — Figure S2. Immune cells infiltrating a Melanoma GVAX vaccine site biopsy are predominantly macrophages. Representative skin biopsy obtained from a patient in Cohort A, 2 days after receiving Cycle 1 of Melanoma GVAX. IHC staining demonstrates a mixed inflammatory infiltrate composed primarily of CD68+ macrophages/histiocytes, with accompanying CD3+ T cells. Melanoma GVAX vaccine cells express the gp100 antigen in situ, as shown by HMB-45 staining. Only rare CD20+ B cells were observed. 200× original magnification, all fields. [file 12967_2015_572_MOESM3_ESM.pptx]
