# Supplementary material for: Safety and immunologic correlates of Melanoma GVAX, a GM-CSF secreting allogeneic melanoma cell vaccine administered in the adjuvant setting
Source: J Transl Med. 2015 Jul 5;13:214. doi: 10.1186/s12967-015-0572-3 (PMC4491237; doi:10.1186/s12967-015-0572-3)
Supplement: Additional file 4: — Figure S3. Trends in peripheral lymphocyte numbers and percentages over time on treatment. Significant decreases in mean peripheral lymphocyte numbers (left panel, p = 0.007) and percentages (right panel, p = 0.018) occurred over time on treatment and follow-up. Linear mixed effect model was used for comparisons. Mean values, represented by solid circles, are connected by a trendline in each graph. Rectangles at each time point extend to the 1st and 3rd quartiles. Vertical lines at each time point extend to 1.5 x the interquartile range. Dashed lines indicate upper and lower limits of normal values. Each treatment cycle length is 28 days. Samples were obtained on Day 1 of each cycle for patients in Cohorts A and B, and on Day 0 of each cycle for patients in Cohort C. An analysis including all patients is shown. Similar trends were seen for each cohort when analyzed individually (data not shown). C, treatment cycle. [file 12967_2015_572_MOESM4_ESM.pptx]

## Slide 1
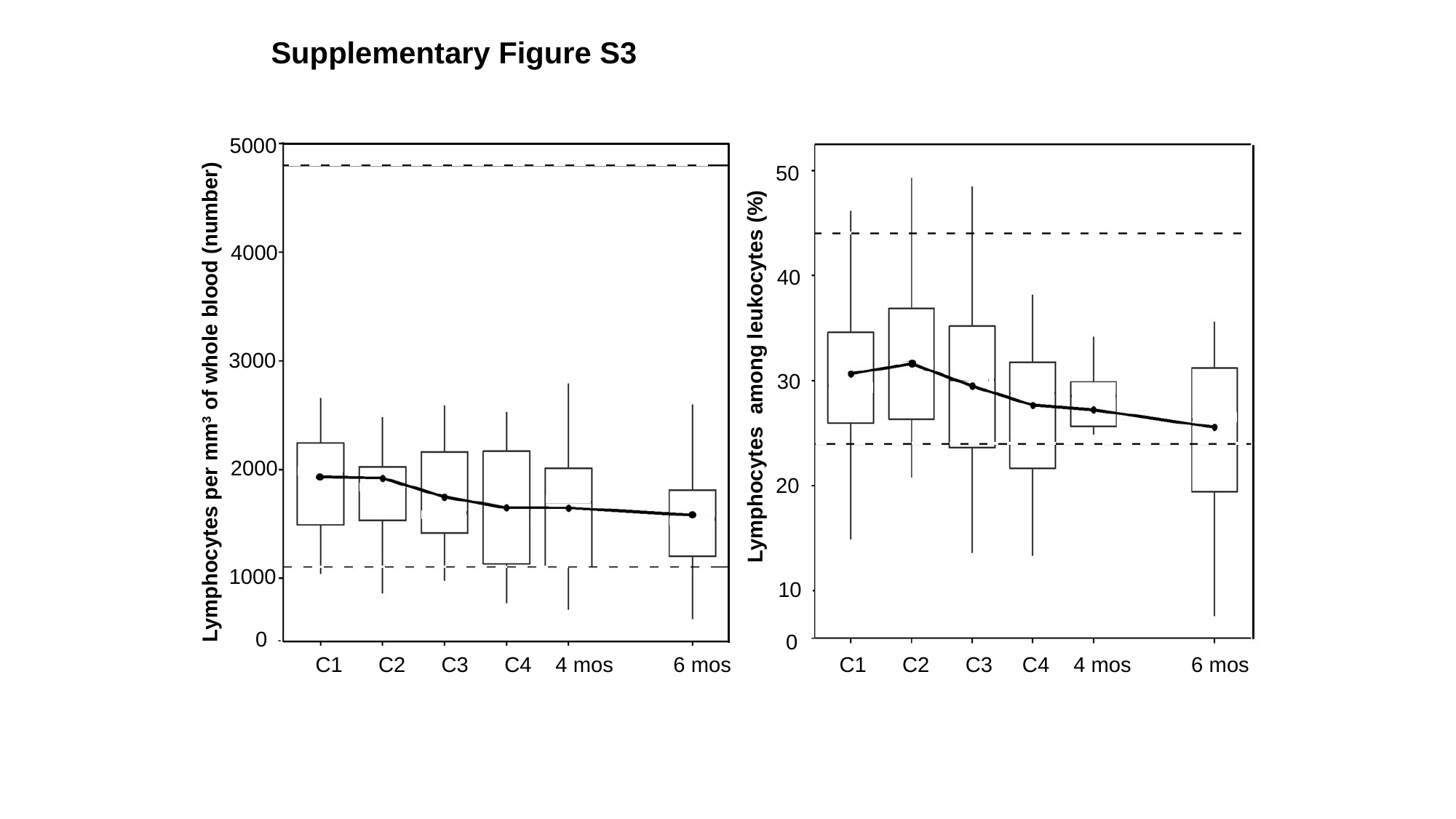

Supplementary Figure S3
5000
50
40
 Lymphocytes among leukocytes (%)
30
20
10
0
 C1 C2 C3 C4 4 mos 6 mos
4000
3000
Lymphocytes per mm3 of whole blood (number)
2000
1000
0
 C1 C2 C3 C4 4 mos 6 mos
