# Supplementary material for: Safety and immunologic correlates of Melanoma GVAX, a GM-CSF secreting allogeneic melanoma cell vaccine administered in the adjuvant setting
Source: J Transl Med. 2015 Jul 5;13:214. doi: 10.1186/s12967-015-0572-3 (PMC4491237; doi:10.1186/s12967-015-0572-3)
Supplement: Additional file 5: — Figure S4. Peripheral blood Treg percentages are not significantly affected by the administration of low-dose CPM prior to Melanoma GVAX inoculation. Right panel: no significant change in the percentage of Tregs (CD4+ , CD25hi, FoxP3 +) among circulating CD4+ T lymphocytes was observed 3 days following CPM 200 mg/m2 IV, in vaccination cycles 1 or 4. Left panel: Treg percentages before and after Melanoma GVAX administration without CPM, in Cohorts A and B. Patients in Cohort C received CPM at D0, and Melanoma GVAX at D1 of each cycle. Bars depict the mean ± SEM. Comparisons were not significant using a paired Wilcoxon signed-rank test. C, treatment cycle; D, treatment day. [file 12967_2015_572_MOESM5_ESM.pptx]

## Slide 1
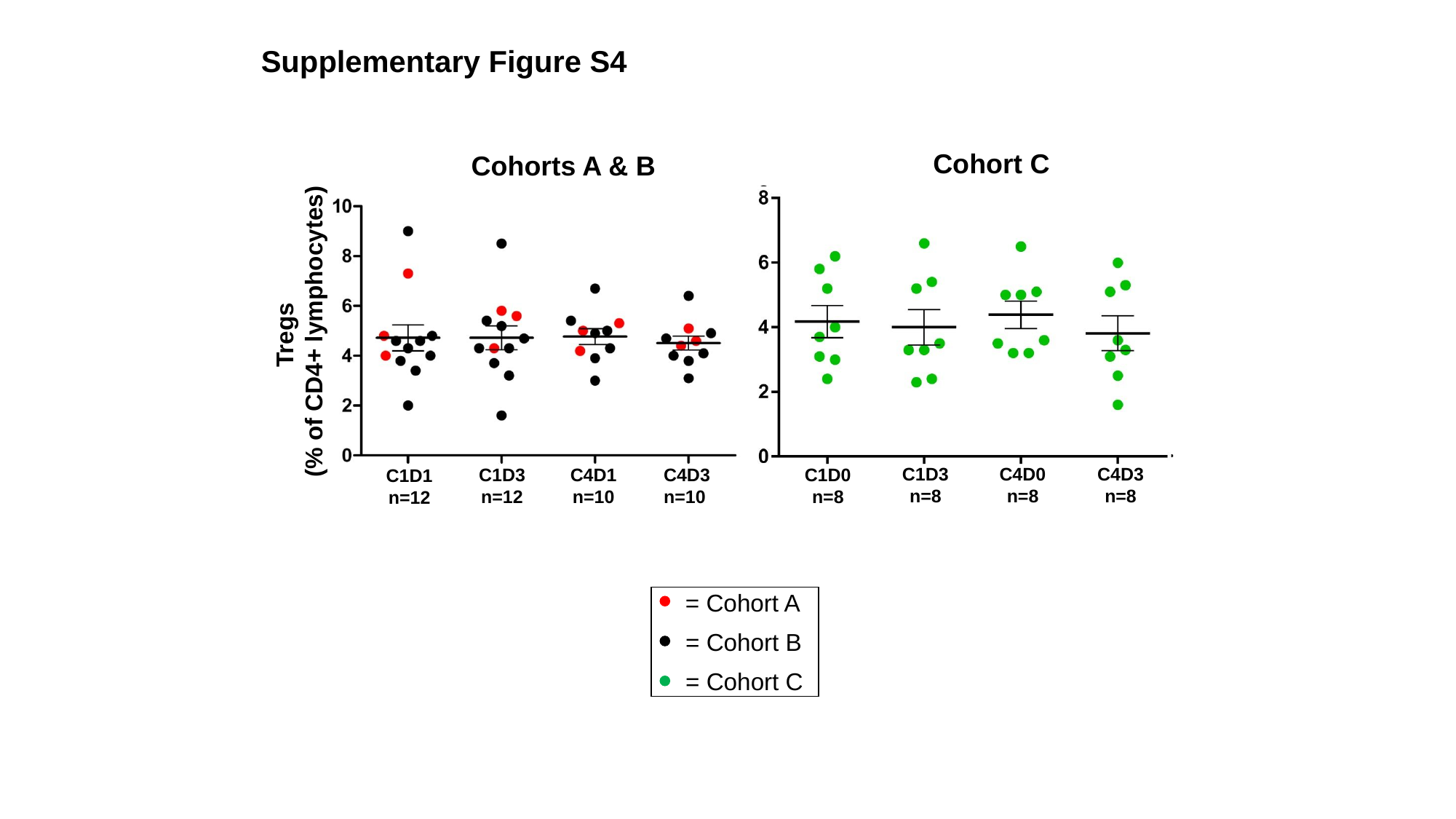

Supplementary Figure S4
Cohort C
Cohorts A & B
Tregs
(% of CD4+ lymphocytes)
C4D3
n=8
C4D0
n=8
C1D3
n=8
C4D3
n=10
C4D1
n=10
C1D3
n=12
C1D0
n=8
C1D1
n=12
= Cohort A
= Cohort B
= Cohort C
